# Meta-analysis of the correlation between personality characteristics and risky driving behaviors

**DOI:** 10.5249/jivr.v11i2.1172

**Published:** 2019-07

**Authors:** Maryam Akbari, B Lankarani Kamran, Seyed Taghi Heydari, Seyed Abbas Motevalian, Reza Tabrizi, Zohreh Asadi-Shekari, Mark J.M.Sullman

**Affiliations:** ^*a*^Health Policy Research Center, Institute of Health, Student Research Committee, Shiraz University of Medical Sciences, Shiraz, Iran.; ^*b*^Department of Epidemiology, School of Public Health, Iran University of Medical Sciences, Tehran, Iran.; ^*c*^Centre for Innovative Planning and Development (CIPD), Faculty of Built Environment, University Teknologi Malaysia.; ^*d*^Department of Social Sciences, University of Nicosia, Cyprus.

**Keywords:** Personality characteristics, Risky driving behaviors, Meta-Analysis

## Abstract

**Background:**

A systematic review and meta-analysis was performed to determine the relationships risky driving behaviors (RDBs) have with the big five personality factors, sensation seeking and driving anger.

**Methods:**

The PubMed, EMBASE, Web of Science, Scopus, Psychinfo, and the Cochrane Library databases were systematically searched. All original studies were retrieved that assessed the relationships RDBs had with the big five personality factors, sensation seeking, and driving anger. Heterogeneity between studies was examined using the Cochran Q statistic and I2 tests. After applying Fisher’s r-to-z transformation, the correlation coefficients (r) were summarized from each study and 95% confidence intervals (CIs) were estimated.

**Results:**

Overall, 22 studies were included in the meta-analysis, which included 11211 participants. The results showed that RDBs had a significant negative relationship with agreeableness (r -0.27; 95% CI, -0.36, -0.19; P less than 0.0001), but significant positive relationships with neuroticism (r 0.16; 95% CI, 0.03, 0.29; P=0.584), sensation seeking (r 0.28; 95% CI: 0.23, 0.33; P less than 0.0001) and driving anger (r 0.39; 95% CI: 0.14, 0.64; P=0.002). Conversely, RDBs were not significantly related to extraversion (r -0.01; 95% CI, -0.08, 0.05; P=0.705), conscientiousness (r -0.05; 95% CI, -0.21, 0.12; P=0.584), or openness (r -0.06; 95% CI, -0.12, 0.00; P=0.065).

**Conclusions:**

Therefore, it appears that individuals most likely to engage in risky driving behaviors would be low in agreeableness, but high in neuroticism, sensation seeking and driving anger.

## Introduction

Risky driving behaviors (RDBs) include behaviors such as: blocking intersections, overtaking on the wrong side, using two lanes, speeding, not using a seat belt, tailgating, driving through an orange light that is turning red, driving without a license, talking on mobile phones, using a hands-free device, double parking, failing to signal, changing lanes without signaling, forcing someone to give way, and weaving in/out of traffic.^1-5^

Previous research has shown that risky driving behaviors are related to collision involvement and greatly increase the chances of injury or death.^6-8^ It has been shown that approximately 40 to 95 percent of road traffic injuries are as a result of risky driving behaviors.^4-5,8-10^ RDBs are a multidimensional in nature and there are many factors that influence engagement in these types of behaviors.^2,11-13^

A driver’s personality is one of the most important underlying causes and a variable strongly relevant to RDBs.^14-15^ For example, one study showed that personality characteristics (PCs) explained more than 35 % of the variance in risky driving behavior.^15^ Of the many PCs identified as potential predictors of RDBs, the big five personality factors (including extraversion, agreeableness, conscientiousness, neuroticism, and openness), sensation seeking, and driving anger have garnered the most support to date.^16^ However, despite the fact that several studies have reported significant correlations (positive/negative) between PCs and RDBs,^16-19^ a number of other studies did not find RBDs were significantly related to PCs, such as: agreeableness,^20^ neuroticism,^6^ extraversion,^20^ openness,^21^ sensation seeking,^22^ and driving anger.^23^ In a meta-analysis conducted by Demir et al.^24^ they reported that driving anger had significant associations with aberrant driving behaviors (based on Driver Behavior Questionnaire - DBQ) factors. In another meta-analysis, using Iranian populations, it was found that more than 50 percent of drivers have sleep quality disorders.^25^ Therefore, the relationships RDBs have with personality factors remains, to some degree, controversial and at the same time are very important. 

Although several studies have examined the influence of personality on RDBs, we are aware of no other systematic review and meta-analysis which attempts to integrate and combine the results and draw conclusions about the effect of PCs (all of the big five personality factors, sensation seeking, and driving anger) on RDBs. The current study was performed to summarize the available evidence to establish the relationships that RDBs have with the big five personality factors, sensation seeking and driving anger.

## Methods

**Search strategy and selection studies**

Eligible studies were identified using PubMed, Embase, Web of Science (WOS), Scopus, Psychinfo, and the Cochrane Library databases for published articles from inception up to December 2017, with two authors (MA & RT) independently searching each database. Also, the reference lists of identified studies were manually searched to increase sensitivity in the search strategy. The search was limited to publications in the English language. The databases were searched using the following keyword search terms: personality [“personality characteristic” OR “personality traits” OR “personality factors” OR “personality variables” OR “extraversion variable” OR “agreeableness variable” OR “conscientiousness variable” OR “neuroticism variable” OR “openness variable” OR “sensation seeking” OR “driving anger”] AND risky driving [“risky driving (RD)” OR “risky driving behaviors (RDBs)”]. 

**Inclusion and exclusion criteria**

Two authors (RT & MA) independently selected all relevant articles, if they met the following inclusion criteria:1) study was original research in the English language; 2) study investigated the correlation between PCs (including extraversion, agreeableness, conscientiousness, neuroticism, openness, sensation seeking, and driving anger) and RDBs; 3) study contained adequate data to calculate the correlation coefficients (r); 4) study used a standard questionnaire for measure personality traits; and 5) the study measured an aspect of risky driving. 

Studies were excluded if they were not published in peer-reviewed journals or did not meet the lowest acceptable quality assessment score. Any disagreements were resolved by discussion and, if required, consensus was reached by consultation with the 3rd author (K B.L).

**Data extraction and quality assessment**

The data were extracted from the eligible studies and the quality of these was assessed in dependently by two authors (RT and MA). The extracted information included the following: 1) first authors’ name, 2) publication year, 3) sample size, 4) gender, 5) age, 6) country of origin, 7) personality measure(s), 9) risky driving measures, 10) personality dimension(s), and 11) the size of the correlation between PCs and RDBs (Pearson’s r, Spearman’s r). If the study did not directly calculate the r, it was computed using the Practical Meta-Analysis Effect Size Calculator.^26^ The quality of the included studies was assessed using a checklist of 12 questions, in accordance with the STROBE checklist.^27-30^ This encompassed various aspects of the methodology, such whether there was an appropriate sample size, study method, sampling, study population, the type of data collection, the variable definitions and sampling method, data collection vehicles, statistical analyses, reporting research findings, and providing results according to the study objectives. A score was allocated to each question and primary studies with at least 8 points were entered into the meta-analysis.^27,31^

**Data analysis**

The r of all primary studies was used to estimate the pooled r between the PCs and RDBs. All types of r were converted to Spearman’s r for the present meta-analysis. Since Spearman’s r is not normally distributed we converted Spearman’s r using Fisher’s r- to -z transformation to achieve z values with an approximately normal distribution and the related 95% confidence interval. The pooled analyses were performed using a random-effects model for meta-analysis. Heterogeneity of effect sizes across studies was examined by calculating Cochran’s Q test and the I2 statistic. An I2 higher than 50 percent with a p-value<0.05 indicated the presence of heterogeneity. Additional analyses, such as subgroup and sensitivity analysis were also performed, when heterogeneity was found. Subgroup analyses were used to examine the source of heterogeneity. Predefined subgroups were produced by age-groups (<20 vs. 20-40 vs. 40 <), gender (female vs. male vs. both), study country (Europe vs. USA vs. other), and personality measures (international personality item pool (IPIP) vs. NEO personality inventory (NEO-IP) vs. other). Sensitivity analyses were conducted to estimate the contribution of each primary study to the pooled r. The existence of potential publication bias was assessed using Egger’s test. Statistical analyses were conducted using STATA version 12.0 software package (Stata Corp., College Station, TX, USA). P-values<0.05 were considered to be statistically significant.

## Results

**Search results**

**Fig 1:**
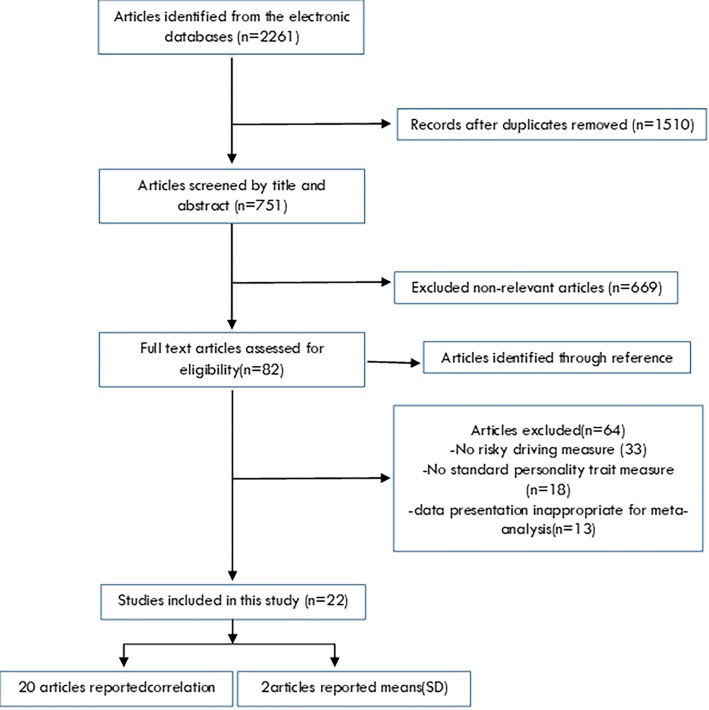
Flowchart is for the selection of eligible studies.

Figure 1 illustrates the details of the study selection process and results according to the preferred reporting items for systematic reviews and meta-analyses guidelines (PRISMA). Finally, after screening, 22 out of the 2261 published studies were included in our meta-analysis.^6,15-23,32-43^ The studies were published between 1994 and 2017. These 22 selected studies included 11211 participants; with a median number of 260 (range: 40-2604) participants per study. Ten studies reported correlation on extraversion, 14 on agreeableness, 17 on conscientiousness, 16 on neuroticism, 8 on openness, 13 on sensation seeking, and 7 on driving anger. The regions of the studies were: 11 from the European continent, 7 from the American continent and 4 from other parts of the world. The personality measures among study participants were the: IPIP in 11 studies, NEO-PI in 4 studies, and other measure in 7 studies. The key characteristics of the studies are presented in Table 1.

**Table 1:** Characteristics of included studies.

Authors (Date)	country	Subjects	Gender	Mean (SD) age of participants	Personality dimension(s)	Personality measures	Risky driving measures
Dahlen et al. (2006)^16^	USA	312 (222 women and 90 men) undergraduate psychology students at the University of Southern Mississippi	both	19 (2.1)	Neuroticism Agreeableness Conscientiousness Openness Extraversion Sensation Seeking anger	International Personality Item Pool (IPIP), Form V of the SSS And Driving Anger Scale (DAS)	Self-reported risky driver (driven without using a seatbelt, passed unsafely, etc.)
Iversen et al. (2002) ^18^	Norway	2604 (1250 men and 1355 women)Norwegian drivers randomly selected from the driver’s licence register	both	45 (15.67)	Conscientiousness Sensation Seeking	Driver Anger Scale (DAS) and SSS Form V	self-completion Driving Behavior Questionnaire (DBQ)
Yang et al. (2013)^15^	China	224 licensed Chinese driver (82 males and 142 females)	both	NR*	Agreeableness (altruism) Conscientiousness (normlessness)sensation-seeking, anger	International Personality Item Pool (IPIP)	completed the Driving Behavior Questionnaire (DBQ) and Ordinary violations
Booth et al. (1994) ^42^	San Diego	103 male U.S. Navy enlisted personnel who were undergoing military basic training	male	19.3 (2.7)	Neuroticism Agreeableness Conscientiousness Openness Extraversion anger	NEO Personality Inventory (NEO-PI) and Driving behaviour scale	completed the traffic risk taking
Machin et al. (2007)^36^	Australia	159 faculties of the University of Southern Queensland (USQ) student population (47 male, and 112 were female)	both	18.8 (1.01)	Altruism Normlessness sensation-seeking	International Personality Item Pool (IPIP)	Speeding scale (Speeding, speed more than 10 km/h,ect)
Deng et al. (2015) ^6^	China	40 students (34 men and 6 women, recruited at Xi’an Jiaotong University.	both	22.8 (2.55)	Neuroticism Extraversion Sensation Seeking	Eysenck Personality Questionnaire (EPQ)	Risk-taking inclination (speeding and competitiveness)
Jovanovic et al. (2011)^37^	Serbia	260 individuals with valid driving licenses completed questionnaires in Serbia (137men and 123 women)	both	32.5 (10.9)	Neuroticism Agreeableness Conscientiousness Openness Extraversion anger	NEO–PI-R scale and Driver Anger Scale (DAS)	aggressive driving
Falco et al. (2013)^23^	Italy	1028 young people in first or second year of high school at their first driving experience(576 were male and 452 were female)	both	14.58 (2.6)	Normlessness Neuroticism Sensation seeking anger	International Personality Item Pool (IPIP), Sensation-Seeking Scale (BSSS) and DAS	Driver Behavior Questionnaire (DBQ) and Ordinary violations
Benfield et al. (2007)^43^	USA	204 undergraduates (85 males and 119 females)	both	18.71 (1.97)	Neuroticism Agreeableness Conscientiousness Openness Extraversion	International Personality Item Pool (IPIP) and (DAS)	aggressive driving
Hartos et al. (2002)^38^	Maryland	261 high schools from adolescents with a driver’s license in two Maryland school districts(115 male,146 women)	both	16.8 (.63)	Sensation seeking	Items from SSS	Exceed the speed limit, Drive through a stop sign, Drive without wearing a safety belt, Drive after drinking alcohol, ect
Seibokaite et al .(2012) ^21^	Lithuania	166 professional drivers (males) who drive small buses and heavy trucks from different Lithuanian organizations	male	41.71 (10.10)	Neuroticism Agreeableness Conscientiousness Openness Extraversion	International Personality Item Pool (IPIP)	Driver Behavior Questionnaire (violations and errors)
Marengo et al. (2012) ^35^	Italy	207 students (108 females, 98 males), attending the first of year of high school in North-East area of Italy.	both	14.5 (.11)	Neuroticism Agreeableness Sensation seeking	Thrill and Adventure Seeking (TAS)	Violations of traffic laws and Driving under the influence of substances
Pearson et al. (2013) ^33^	USA	266 college student drivers (162 women, 104 men))	both	22.75 (6.32)	Sensation seeking	Thrill and Adventure Seeking (TAS)	Completed the Driving Behavior Questionnaire (DBQ)
Chraif et al. (2015)^19^	Romania	293 drivers selected from two auto services Companies.(252 were male and 41 female)	both	31.34 (8.57)	Neuroticism Agreeableness Conscientiousness Openness Extraversion anger	International Personality Item Pool (IPIP), and (DAS)	Aggressive driving
Constantinou et al. (2011) ^39^	Cyprus	352 young adults were white, Greek-Cypriots ( 241 male, 109 female)	both	20.29 (1.59)	Neuroticism Sensation seeking	(BIS11) and Form (SSS-V)	Driving Behavior Questionnaire (DBQ) and Ordinary Violations
Oltedal et al. (2006) ^34^	Norway	1356 high school classes within Norwegian counties( 724 women , 632 men)	both	18.5 (.12)	Neuroticism Conscientiousness Sensation seeking anger	NEO–PI-R scale and(DAS)	termed speeding, rule violations and self-assertiveness
Qu et al. (2015) ^17^	China	295 licensed Chinese drivers through interviewing people around parking lots or residential quarters(148 males and 147 females)	both	37.34 (9.39)	Neuroticism Agreeableness Conscientiousness	International Personality Item Pool (IPIP)	Risky Driving, Aggressive Driving, Negative Cognitive/Emotional Driving and Drunk Driving
Burtaverde et al. (2017) ^40^	Romania	244 driver community respondents (178 women,66 men)	both	26.75 (8.27)	Neuroticism Agreeableness Conscientiousness Openness Extraversion	International Personality Item Pool (IPIP)	Enjoy the excitement of dangerous driving
Ulleberg et al. (2003) ^32^	Norway	1881adolescents in Norway (1053 were women and 828 were men)	both	18.5 (1.8)	Agreeableness Conscientiousness Sensation seeking	NEO-Personality Inventory	risk-taking in traffic (speeding, rule violations and self-assertiveness)
Sween et al. (2017) ^20^	Italy	804 Italian community sample(466 female.338 men)	both	34.96 (8.25)	Neuroticism Agreeableness Conscientiousness Openness Extraversion	HEXACO-PI-R	Risk Taking (Mobile phone use while driving)
Schwebel et al. (2006) ^22^	USA	73 college students from introductory psychology courses at the University of Alabama at Birmingham(31 male, 42 female)	both	27.82 (7.94)	Neuroticism Conscientiousness Sensation seeking	International Personality Item Pool (IPIP), SSS-V	Driving Behavior Questionnaire (DBQ)(violations, speed)
Brown et al. 2016^41^	Canada	83 adult male drivers	male	30 (5.7)	Neuroticism Agreeableness Conscientiousness Openness Extraversion	International Personality Item Pool (IPIP)	driving while impaired group (DWI)

*NR: non reported , both; male and female.

**Main outcomes**

**Fig 2:**
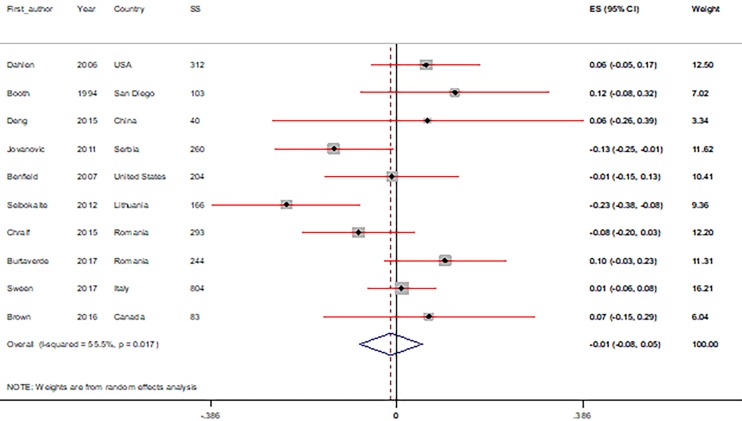

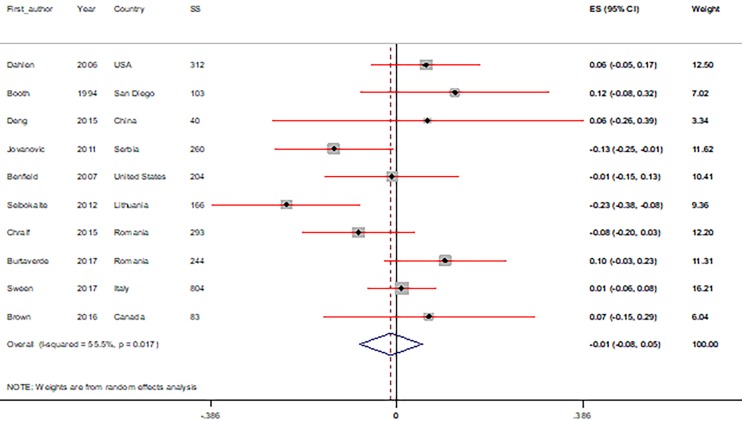

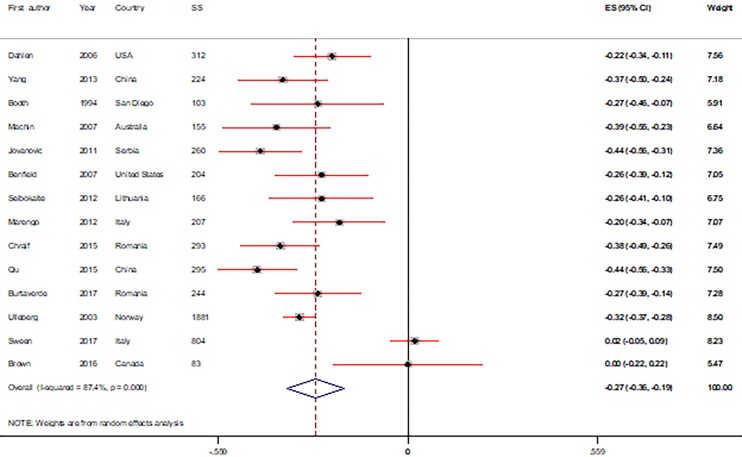

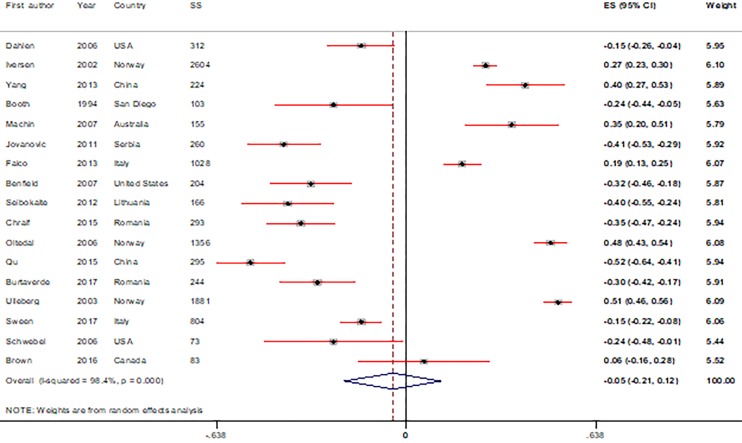

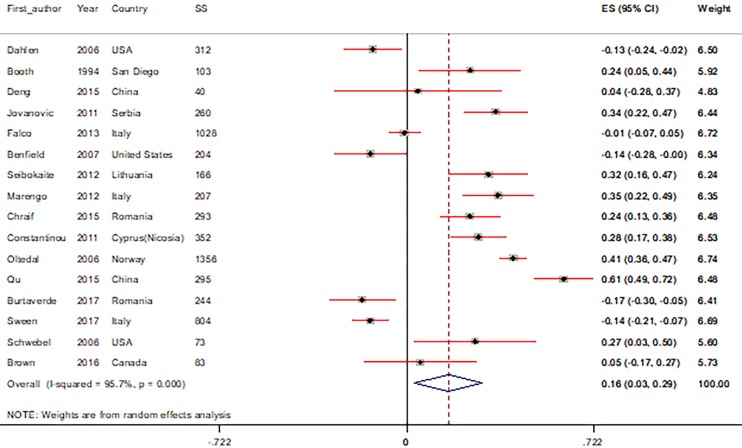
Meta-analysis correlation coefficient estimates between the big five personality factors, including: (A) extraversion, (B) for agreeableness, (C) for conscientiousness, (D) for neuroticism, (E) and for openness with risky driving behaviors (CI=95%).

**Fig 3:**
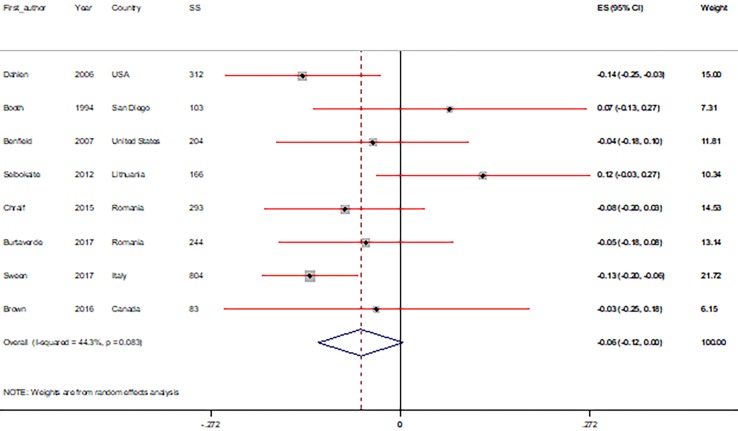

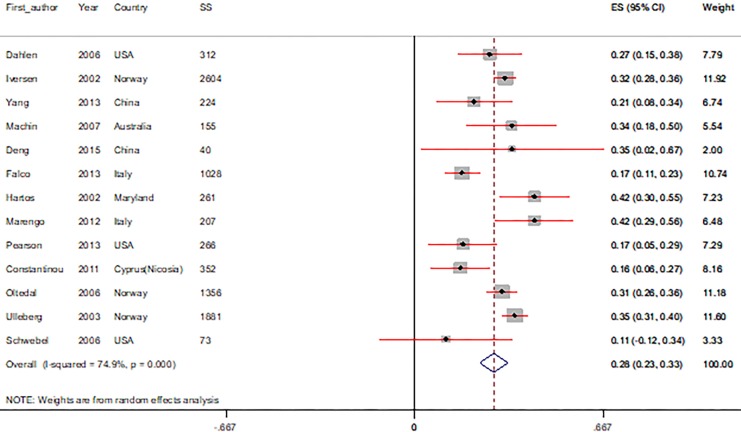
Meta-analysis correlation coefficient estimates between (A) sensation seeking and (B) for driving anger with risky driving behaviors (CI=95%).

Pooled estimates of the r between PCs (big five personality factors, sensation seeking, and driving anger) and RDBs are shown in Figure 2 and Figure 3. The correlations between PCs and RDBs, based on the subgroup and sensitivity analysis, are shown in Tables 2 and 3. The results of the subgroup and sensitivity analyses showed that the correlations were different in some of the specific subgroups for the measured variables and in each study.

**Table 2:** The correlation between personality characteristics and risky driving behaviors, based on subgroup analysis.

Variable		Number of SMD included	Subgroups	Pooled effect estimate	95% CI	I2 (%)	Overall I2 (%)
**Extraversion**		4	America	0.05	-0.03, 0.12	0.0	
	Continent	5	Europe	-0.06	-1.16, 0.04	73.6	
		1	Other	0.06	-0.26, 0.39	-	
		6	IPIP	-0.02	-0.11, 0.08	64.9	
	Personality measures	2	NEO-PI	-0.02	-0.26, 0.23	78.0	
		2	Other	0.01	-0.06, 0.08	0.0	55.5
		3	<20	0.05	-0.03, 0.13	0.0	
	Age groups	6	20-40	-0.01	-0.08, 0.06	45.0	
		1	40<	-0.23	-0.38, -0.08	-	
		-	Female	-	-	-	
	Gender	3	Male	-0.02	-0.26, 0.21	78.4	
		7	Both	-0.00	-0.06, 0.05	40.2	
**Agreeableness**		4	America	-0.21	-0.30, -0.11	30.4	
	Continent	7	Europe	-0.26	-0.39,-0.13	92.7	
		3	Other	-0.41	-0.48, -0.33	0.00	
		9	IPIP	-0.30	-0.37, -0.23	59.6	
	Personality measures	3	NEO-PI	-0.34	-0.42, -0.26	41.8	
		2	Other	-0.08	-0.30, 0.13	87.6	87.4
		6	<20	-0.29	-0.34, -0.24	19.6	
	Age groups	6	20-40	-0.25	-0.45, -0.06	94.0	
		1	40<	-0.26	-0.41, -0.10	-	
		-	Female	-	-	-	
	Gender	3	Male	-0.19	-0.34, -0.03	51.7	
		11	Both	-0.29	-0.39, -0.20	89.7	
**Conscientiousness**		5	America	-0.19	-1.31, -0.07	57.4	
	Continent	9	Europe	-0.01	-0.22, 0.19	98.8	
		3	Other	0.08	-0.56, 0.71	98.5	
		11	IPIP	-0.12	-0.31, 0.08	96.4	
	Personality measures	4	NEO-PI	0.09	-0.24, 0.43	98.8	
		2	Other	0.06	-0.35, 0.47	99.1	98.4
		7	<20	0.12	-0.09, 0.34	98.0	
	Age groups	7	20-40	-0.29	-0.42, -0.16	87.0	
		2	40<	-0.06	-0.71, 0.59	98.5	
	-	Female	-0.80	-1.76, 0.16	85.9		
	Gender	3	Male	-0.20	-0.46, 0.05	82.2	
		14	Both	-0.01	-0.19, 0.16	98.6	
**Neuroticism**		5	America	0.04	-0.13, 0.21	79.8	
	Continent	9	Europe	0.18	0.01,0.34	96.7	
		2	Other	0.16	0.03, 0.29	95.7	
		9	IPIP	0.11	-0.06, 0.29	94.6	
	Personality measures	3	NEO-PI	0.37	0.29, 0.45	39.7	
		4	Other	0.14	-0.15, 0.42	95.4	95.7
		6	<20	0.12	-0.10, 0.34	97.0	
	Age groups	9	20-40	0.17	-0.03, 0.37	95.4	
		1	40 <	0.32	0.16, 0.47	-	
		-	Female	-	-	-	
	Gender	3	Male	0.22	0.07, 0.37	46.5	
		13	Both	0.15	0.00, 0.30	96.5	
**Openness**		4	America	-0.06	-0.15, 0.03	21.0	
	Continent	4	Europe	-0.05	-0.15, 0.04	65.5	
		-	Other	-	-	-	
		6	IPIP	-0.05	-0.12, 0.02	33.0	
	Personality measures	1	NEO-PI	0.07	-0.13, 0.27	-	
		1	Other	-0.13	-0.20, -0.06	-	89.9
		3	< 20	-0.06	-0.17, 0.06	45.8	
	Age groups	4	20-40	-0.10	-0.15, -0.05	0.0	
		1	40 <	0.12	-0.03, 0.27	-	
		-	Female	--	-	-	
	Gender	3	Male	0.07	-0.04, 0.17	0.0	
		5	Both	-0.10	-0.15, -0.06	0.0	
**Sensation seeking**		4	America	0.26	0.13, 0.39	71.2	
	Continent	6	Europe	0.29	0.22, 0.35	85.5	
		3	Other	0.27	0.17, 0.37	0.0	
		5	IPIP	0.22	0.15, 0.28	34.4	
	Personality measures	2	NEO-PI	0.33	0.29, 0.38	36.0	
		6	Other	0.30	0.21, 0.39	73.7	74.9
		7	< 20	0.32	0.25, 0.39	80.7	
	Age groups	4	20-40	0.17	0.10, 0.24	0.0	
		1	40 <	0.32	0.28, 0.36	-	
		-	Female	-	-	-	
	Gender	-	Male	-	-	-	
		13	Both	0.28	0.23, 0.33	74.9	
**Driving anger**		1	America	0.32	0.21, 0.43	-	
	Continent	4	Europe	0.44	0.05, 0.83	99.0	
		2	Other	0.31	0.21, 0.41	0.0	
		5	IPIP	0.42	0.01, 0.82	98.6	
	Personality measures	2	NEO-PI	0.32	0.06, 0.58	93.5	
		-	Other	-	-	-	98.0
		4	< 20	0.20	0.06, 0.35	91.8	
	Age groups	2	20-40	0.79	0.14, 1.0	98.4	
		-	40 <	-	-	-	
		-	Female	-	-	-	
	Gender	-	Male	-	-	-	
		7	Both	0.39	0.14, 0.64	98.0	
**Openness**		4	America	-0.06	-0.15, 0.03	21.0	
	Continent	4	Europe	-0.05	-0.15, 0.04	65.5	
		-	Other	-	-	-	
		6	IPIP	-0.05	-0.12, 0.02	33.0	
	Personality measures	1	NEO-PI	0.07	-0.13, 0.27	-	
		1	Other	-0.13	-0.20, -0.06	-	89.9
		3	< 20	-0.06	-0.17, 0.06	45.8	
	Age groups	4	20-40	-0.10	-0.15, -0.05	0.0	
		1	40 <	0.12	-0.03, 0.27	-	
		-	Female	-	-	-	
	Gender	3	Male	0.07	-0.04, 0.17	0.0	
		5	Both	-0.10	-0.15, -0.06	0.0	
**Sensation seeking**		4	America	0.26	0.13, 0.39	71.2	
	Continent	6	Europe	0.29	0.22, 0.35	85.5	
		3	Other	0.27	0.17, 0.37	0.0	
		5	IPIP	0.22	0.15, 0.28	34.4	
	Personality measures	2	NEO-PI	0.33	0.29, 0.38	36.0	
		6	Other	0.30	0.21, 0.39	73.7	74.9
		7	< 20	0.32	0.25, 0.39	80.7	
	Age groups	4	20-40	0.17	0.10, 0.24	0.0	
		1	40 <	0.32	0.28, 0.36	-	
		-	Female	-	-	-	
	Gender	-	Male	-	-	-	
		13	Both	0.28	0.23, 0.33	74.9	
**Driving anger**		1	America	0.32	0.21, 0.43	-	
	Continent	4	Europe	0.44	0.05, 0.83	99.0	
		2	Other	0.31	0.21, 0.41	0.0	
		5	IPIP	0.42	0.01, 0.82	98.6	
	Personality measures	2	NEO-PI	0.32	0.06, 0.58	93.5	
		-	Other	-	-	-	
		4	< 20	0.20	0.06, 0.35	91.8	98.0
	Age groups	2	20-40	0.79	0.14, 1.0	98.4	
		-	40 <	-	-	-	
	-	Female	-	-	-		
	Gender	-	Male	-	-	-	
		7	Both	0.39	0.14, 0.64	98.0	

**Table 3:** Sensitivity analysis of the correlation between personality characteristics and risky driving behaviors.

Parameter	Pre-sensitivity analysis			Upper & lower of effect size	Post-sensitivity analysis		
	No. of Studies included	Pooled r(random effect)	95% CI		Pooled r(random effect)	95% CI	Excluded studies
****				Upper	0.007	-0.04, 0.06	Seibokaite^21^
**Extraversion**	10	-0.01	-0.08, 0.05				
****				Lower	-0.02	-0.09, 0.03	Burtaverde^40^
****				Upper	-0.25	-0.34, -0.17	Qu^17^
**Agreeableness**	14	-0.27	-0.36, -0.19				
****				Lower	-0.30	-0.35, -0.25	Sween^20^
****				Upper	-0.01	-0.17, 0.14	Qu^17^
**Conscientiousness**	17	-0.05	-0.21, 0.12				
****				Lower	-0.08	-0.24, 0.08	Ulleberg^32^
****				Upper	0.18	0.05, 0.31	Burtaverde^40^
**Neuroticism**	16	0.16	0.03, 0.29				
****				Lower	0.13	0.003, 0.25	Qu^17^
****				Upper	-0.03	-0.10, 0.02	Sween^20^
**Openness**	8	-0.06	-0.12, 0.00				
****				Lower	-0.09	-0.13, -0.04	Seibokaite^21^
****				Upper	0.29	0.25, 0.34	Falco^23^
**Sensation seeking**	13	0.28	0.23, 0.33				
****				Lower	0.26	0.21, 0.31	Hartos^38^
****				Upper	0.45	0.17, 0.73	Falco^23^
**Driving anger**	7	0.39	0.14, 0.64				
****				Lower	0.26	0.12, 0.39	Chraif^19^

Abbreviation: r; correlation coefficient.

**Correlation between the big five personality factors and RDBs **

The correlations between RDBs and the big five personality factors are shown in Figure 2 . Meta-analysis of the data showed a significant negative relationship between RDBs and agreeableness (r -0.27; 95% CI, -0.36, -0.19; P <0.0001), while neuroticism had a significant positive relationship (r 0.16; 95% CI, 0.03, 0.29; P=0.584). There was no significant relationship between RDBs and extraversion (r -0.01; 95% CI, -0.08, 0.05; P=0.705), conscientiousness (r -0.05; 95% CI, -0.21, 0.12; P=0.584), or openness (r -0.06; 95% CI, -0.12, 0.00; P=0.065). 

Similarly, in subgroup analyses we found a significant relationship between RDBs and agreeableness. However, in the subgroup analysis by personality measures, the other category (r -0.08; 95% CI, -0.30, 0.13; P=0.454) was not significant, while the IPIP (r -0.30; 95% CI, -0.37, -0.23; P<0.0001) and NEO-IP (r -0.34; 95% CI, -0.42, -0.26; P <0.0001) categories were both significant. Neuroticism was not significantly related to RDBs for: the American continent (r 0.04; 95% CI, -0.31, 0.21; P=0.634), personality measures using the IPIP (r 0.11; 95% CI, -0.06, 0.29; P=0.204), the other category (r 0.14; 95% CI, -0.15, 0.42; P=0.352), those aged <20 (r 0.12; 95% CI, -0.10, 0.34; P=0.282) or 20-40 (r 0.17; 95% CI, -0.03, 0.37; P=0.087) years old. Nevertheless, all of these categories for neuroticism had a positive relationship with RDBs. Details of the subgroup analyses for the other factors (extraversion, conscientiousness, openness) are shown in Table 2. 

In the sensitivity analysis, to determine the effect of each study on the strength of the relationship between big five personality factors and RDBs, the pooled r were estimated after excluding each study from the analysis. This meta-analysis showed no significant difference between the pre- and post-sensitivity pooled rs, but for openness there were significant differences between pre -0.06 (95% CI: -0.12, 0.00) and post-sensitivity pooled r-0.09 (95% CI: -0.13, -0.04), after omitting the Seibokaite et al. article^21^ (Table 3).

**Correlation between sensation seeking and driving anger with RDBs**

Similar findings were observed for sensation seeking and driving anger. A significant positive correlation of r 0.28 (95% CI: 0.23, 0.33; P<0.0001) was found between RDBs and sensation seeking, while the relationship between RDBs and driving anger found a significant positive relationship of r 0.39 (95% CI: 0.14, 0.64; P=0.002) ((Fig. 3). In the subgroup analyses, we found that the significant positive relationships that sensation seeking and driving anger had with RDBs were not influenced by continent, personality measures, age groups, or gender (Table 2). Sensitivity analyses were conducted, and the findings for sensation seeking and driving anger remained consistent with the pooled r. The lower and higher pooled r in the sensitivity analysis for sensation seeking were 0.26 (95% CI: 0.21, 0.31), after omitting the Hartos et al.^38^ and 0.29 (95% CI: 0.25, 0.34) after omitting Falco et al., ^23^ respectively. For driving anger, a lower pooled r was found in the sensitivity analysis of 0.26 (95% CI: 0.12, 0.39) after omitting the Chraif et al^19^ and a higher pooled r of 0.45 (95% CI: 0.17, 0.73) after omitting Falco et al. ^23^ (Table 3). 

**Publication Bias**

Egger’s regression was performed to detect potential publication bias among the studies included in the meta-analysis. Egger’s regression revealed no significant publication bias for the relationships RDBs had with extraversion (B=0.05, P= 0.969), agreeableness (B=-0.39, P= 0.830), neuroticism (B=-0.17, P= 0.954), sensation seeking (B=-0.91, P= 0.416), or driving anger (B=9.84, P= 0.173). 

Because there was evidence of publication bias for conscientiousness (B = -9.95, P = 0.004) and openness (B= 2.78, P = 0.030), non-parametric analyses were performed (Duval and Tweedie). The meta-analysis based on the censored studies indicated that the pooled r on conscientiousness was not significantly changed before -0.05(95% CI, -0.21, 0.12) or -0.05 (95% CI, -0.21, 0.12) after the censored studies were included in the meta-analysis. For openness, the analysis indicated that the pooled r on openness significantly changed before -0.06 (95% CI, -0.12, 0.00) and after -0.09 (95% CI, -0.16, -0.03) when the censored studies were included in the meta-analysis. The finding for openness approximately agreed with what we expected. Therefore, according to the results of the Egger’s regression tests the current findings were supported.

## Discussion

As far as the authors are aware, this systematic review and meta-analysis is the first to study the relationships the big five personality factors (including extraversion, agreeableness, conscientiousness, neuroticism, and openness), sensation seeking, and driving anger have with RDBs among drivers from around the world. Our meta-analysis indicated that the relationship RDBs have with agreeableness was negative, and with neuroticism, sensation seeking, and driving anger there were positive relationships. In contrast, RDBs were not significantly related to extraversion, conscientiousness, or openness.

The finding of a negative relationship with agreeableness has been supported by previous researches, which have demonstrated that risky driving behavior can be predicted by agreeableness. These studies have reported that low levels of agreeableness can predict high levels of risky driving outcomes, including crashes.^15-17,19,21,32,35-37,40,42-43^ In contrast, research by Brown et al. (2016) and Sween et al. (2017) found no significant relationships between Agreeableness and RDBs.^20,41^ There may be a number of reasons for these disparate findings. The small sample size in Brown et al. or the different personality measures used in Sween et al. may explain the discrepancies with the current findings.

This meta-analysis of primary studies also showed that neuroticism was positively related to RDBs, meaning that higher levels of neuroticism were related to higher level of RDBs and vice versa. This finding has been supported by several studies which have shown that those high in neuroticism show more risky driving behaviours.^17,19,21,34-35,37,39,42^ In contrast, two studies reported that neuroticism had no relationship with RDBs.^6,41^ The most likely reason for this discrepancy may be that these studies did not have sufficient sample sizes to answer the questions being studied. Furthermore, a small number of studies have reported that neuroticism had a significant negative relationship with RDBs.^16,40,43^ Perhaps this dissimilar finding was due to the mean age of participants in these studies, which mainly consisted of young adults. Our subgroup analysis indicated that in the >40 years old age group RDBs were positively related to neuroticism. Therefore, this personality factor is particularly important when attempting to reduce risky driving behaviors in this age group.

Similar to previous studies, our meta-analysis confirmed that sensation-seeking and driving anger had significant positive relationships with risky driving behaviours.^15-16,34,36^ Johan et al. also found that persons with high levels of sensation-seeking perform risky driving behaviors to satisfy their need for strong emotions, despite perceiving the risk associate with some risky behaviours.^14^ Consequently, individuals with high levels of sensation–seeking are exposed to an elevated driving risk, so effective interventions need to be investigated in future studies.

Previous research on driving anger has found significant positive relationships with risky driving behaviors, including losing control of one’s vehicle.^16^ Previous research has also shown that driving anger is common. ^44^ However, we need to investigate relevant interventions to deal with driving related anger. In research conducted by Deffenbacher et al. cognitive and physical relaxation interventions were found to significantly reduce risky driving behaviours. ^45^

**Strengths and limitations**

This study, like other study, has some potential strengths and limitations. Firstly, although the number of participants included in our meta-analysis was large, according to the subgroup analysis there were limitations with certain subgroups, which may limit our ability to generalize our findings. Secondly, the present study only included published articles that reported data we could use to estimate the pooled r, which resulted in the exclusion of many other studies. However, Egger’s test indicated no evidence of publication bias among the included studies and a random effects model was used to decrease the heterogeneity effects on the pooled r. Thus, the present study’s findings appear to be reliable.

## Conclusion

Overall, despite these limitations the current meta-analysis demonstrated that the relationship between RDBs and agreeableness was negative, and with neuroticism, sensation seeking, and driving anger there were positive correlations. Given these findings, we need to pay more attention to the importance of traffic psychology in order to reduce and control risky driving behaviors. An additional prospective study with a larger sample size is warranted to investigate these RDBs in the presence of personality dimensions.
